# Suicides disguised as homicides through weapon concealment: A two‐way copycat phenomenon between fiction and real world

**DOI:** 10.1111/1556-4029.70431

**Published:** 2026-08-10

**Authors:** Ernesto Damiani, Alessia Comacchio, Andrea Porzionato

**Affiliations:** ^1^ Department of Medicine – DIMED University of Padova Padova Italy; ^2^ Operational Unit of Forensic Medicine INPS – Padova Padova Italy; ^3^ Section of Human Anatomy, Department of Neuroscience University of Padova Padova Italy

**Keywords:** copycat phenomenon, crime fiction, forensic pathology, forensic staging, load‐and‐rope, narrative transmission, suicide disguised as homicide, weapon concealment

## Abstract

Suicides disguised as homicides present a persistent challenge in forensic case analysis, particularly when the weapon is deliberately removed after discharge. This study investigates the evolution of weapon concealment techniques—specifically the “load‐and‐rope” and “helium balloon‐tied gun” methods—across documented casework, forensic literature, and detective fiction. Through comparative textual analysis and a review of suicides staged as homicides from 1893 to the present, we identify a recurring forensic staging pattern that links narrative transmission to real‐world behavior and vice versa. The “load‐and‐rope” trick, first described in 1893 by Hans Gross, re‐emerged in detective fiction beginning in 1922 before resurfacing in real forensic cases. The “helium balloon” method, introduced in a 2003 episode of *CSI: Crime Scene Investigation*, was subsequently replicated in two confirmed suicides. Based on these findings, we propose an interpretation rooted in the copycat phenomenon, whereby fictional forensic staging techniques—diffused through literature and media—are adopted and adapted in actual suicides. Recognizing this narrative transmission is essential for forensic professionals tasked with distinguishing suicides from homicides. This study underscores the need for interdisciplinary awareness in forensic staging, where cultural narratives may shape criminal behavior as much as technical means.

**Highlights**

- Suicides staged as homicides via weapon concealment are documented from 1893 to the present.
- Load‐and‐rope and helium balloon are identified as recurring weapon concealment techniques.
- Mutual influences and copycat phenomena may be identified between real‐world cases and fiction.
- Practitioners should be aware of narrative transmission potentially shaping forensic death investigations.

## 1 INTRODUCTION

Suicides that are deliberately designed to resemble homicides pose a unique challenge in forensic investigation, particularly when the weapon is removed or concealed after discharge. In gunshot suicides, the presence of the firearm is a key indicator of self‐infliction; its absence typically triggers a homicide inquiry, complicating forensic case analysis and delaying accurate conclusions [1]. Although the forensic literature has occasionally documented such cases, most accounts remain anecdotal and scattered. Conversely, suicides staged as homicides through weapon concealment mechanisms are also present in literary works and fiction, suggesting potential mutual influences. Two specific techniques have emerged as recurring patterns in both forensic casework and narrative sources: the “load‐and‐rope” method, in which the firearm is mechanically retrieved after discharge, and the “helium balloon‐tied gun” method, in which the weapon is carried away by a balloon. This article aims to trace the origins and transmission of such staging techniques by examining their documented forensic instances, fictional representations in detective literature and television, and the potential influence of narrative exposure on suicidal behavior. By analyzing historical and contemporary cases, the study explores whether these suicides reflect original invention or a copycat phenomenon rooted in cultural and media narratives. The goal is to provide forensic professionals with a deeper understanding of how narrative transmission may shape crime scene deception and complicate the distinction between suicide and homicide.

## 2 MATERIALS AND METHODS

This study adopts a qualitative, historical‐comparative design aimed at tracing across forensic literature, fiction, and documented casework the narrative transmission of techniques of disguising suicides as homicides. Primary sources were selected based on their relevance to suicides deliberately staged to resemble homicide through mechanical removal or concealment of the weapon. Inclusion criteria focused on cases involving weapon concealment—defined as firearms displaced post‐discharge via elastic tension, gravity, buoyancy, or other mechanical means. Sources not meeting these criteria were excluded. The corpus comprises 15 sources spanning from 1893 to 2018, including 1 historical documentation, 7 fictional adaptations, and 6 documented forensic cases. It includes forensic manuals, peer‐reviewed forensic literature, detective fiction, television portrayals, and films, including crime dramas and thrillers that depict suicides staged to resemble homicide, with particular attention to scenes involving weapon concealment or forensic deception.

A comparative textual analysis was conducted to identify recurring forensic staging motifs and to examine how fictional representations may influence real‐world forensic behavior. The analysis focused on narrative structure, mechanical plausibility, and forensic framing across sources. Particular attention was given to the presence of weapon concealment mechanisms and the mimicking of homicide through suicide.

Source selection and case inclusion were systematically documented to ensure transparency and reproducibility. Limitations include the reliance on publicly available case reports and fictional texts, which may not reflect the full scope of forensic practice.

## 3 RESULTS

### 3.1 Forensic origins: Hans Gross and the beginning of the load‐and‐rope trick

The earliest documented case of firearm removal via a counterweight mechanism was published in 1893 by Hans Gross (1847–1915) in his *Handbuch für Untersuchungsrichter* [2]. Gross recounts the case of a bankrupt grain merchant who staged his death to resemble homicide in order to secure insurance benefits. The victim was found on a bridge with signs of a struggle and missing valuables. A fresh mark on the railing prompted investigators to search the river below, where they recovered a pistol tied to a stone—rigged to be pulled underwater after discharge. Experimental replication confirmed the setup's effectiveness, demonstrating a deliberate attempt to mimic murder through mechanical staging.

In his final edition of the handbook [3], Gross expanded on the concept of “dissimulated suicide,” defining it as a suicide concealed to appear as natural death, accident, or homicide. He identified various motivations for such concealment, including religious beliefs, social stigma, and financial incentives—particularly the circumvention of pension or insurance exclusions. The 1893 case was reprinted in this edition, underscoring its forensic relevance.

To the best of our knowledge, no other documented case involving mechanical removal of a firearm appeared in the forensic literature up to the second half of the 20th century. However, in this temporal gap, examples of weapon removal as a disguising method are present in fiction.

### 3.2 Fictional revivals of the 1920s: The load‐and‐rope trick in detective literature and film

In particular, the “load‐and‐rope” trick resurfaced in popular culture during the 1920s in detective fiction. These fictional revivals suggest a narrative transmission of forensic staging techniques that may have influenced later real‐world suicides.

#### 3.2.1 The problem of Thor bridge by Arthur Conan Doyle

In *The Problem of Thor Bridge* (1922), Sir Arthur Conan Doyle reimagines Gross's scenario [4]. The story features a woman who stages her suicide as murder by tying a revolver to a rock and allowing it to be pulled into the river after discharge. While Gross's original case involved insurance fraud, Conan Doyle shifts the motive to revenge. A striking textual parallel supports the hypothesis of direct influence. The original publication of Doyle's story includes an illustration (Figure 1) showing Holmes kneeling beside the parapet, examining it for forensic clues. In the narrative, he identifies “a perfectly fresh chip just opposite the body” on the bridge's stonework—an observation that closely echoes Gross's 1906 description of “a small but perfectly fresh injury on the decayed wooden parapet” [3] (Table 1). This forensic clue, central to both narratives, reinforces the continuity between real and fictional staging logic. A famous television adaptation of *The Problem of Thor Bridge*, starring Jeremy Brett, aired in 1991 (Figure 2).

**FIGURE 1:**
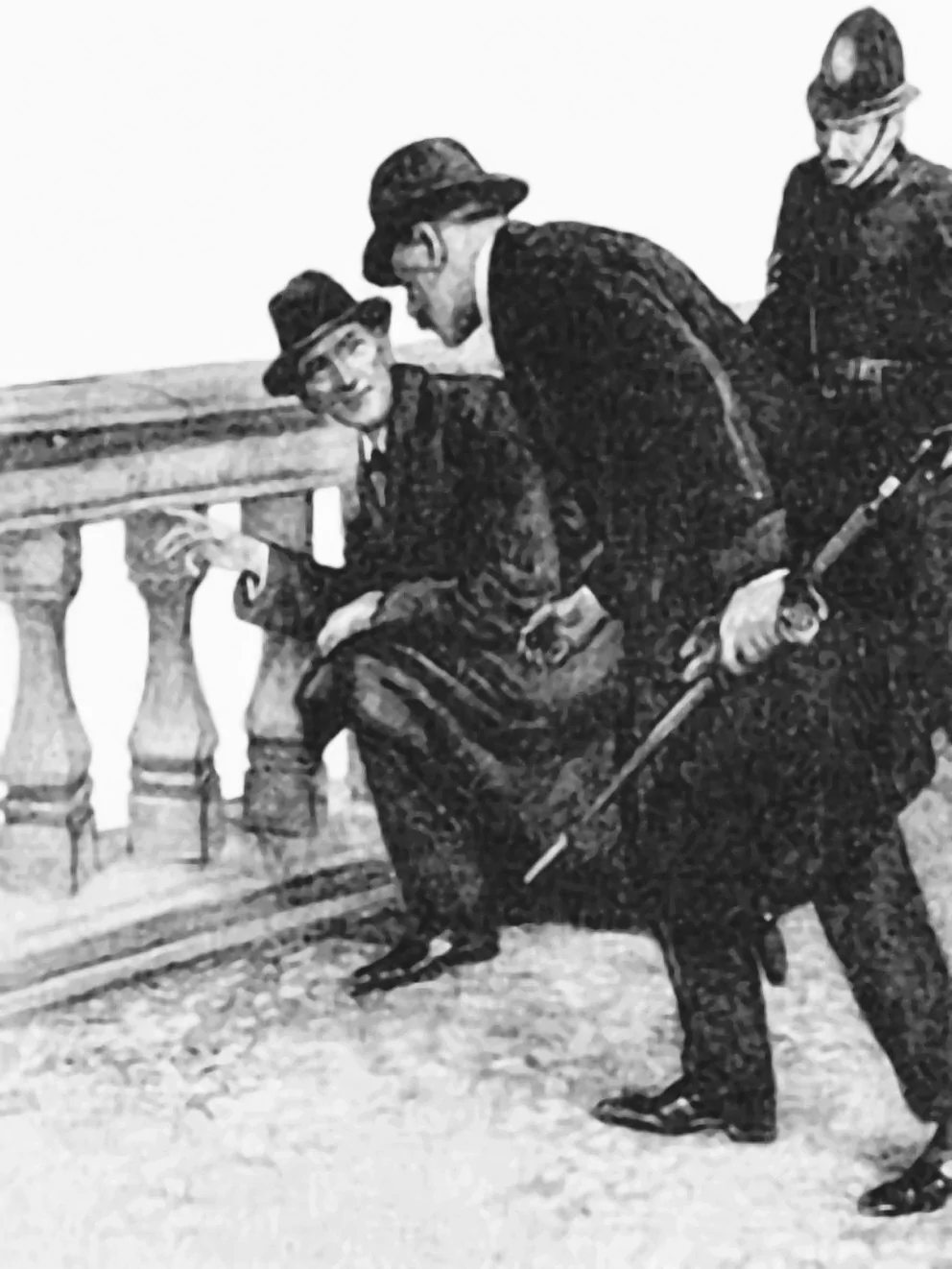
Illustration by Alfred Gilbert from *The Problem of Thor Bridge* by Arthur Conan Doyle, originally published in *The Strand Magazine* (1922) [4]. This image is in the public domain. The image depicts Sherlock Holmes examining a fresh mark on the bridge parapet—an investigative clue central to solving the case. This visual detail echoes Hans Gross's 1893 forensic case [2].

**TABLE 1:** Side comparison of Hans Gross's forensic description (1893) and Arthur Conan Doyle's fictional rendering (1922) of a parapet mark left by a weapon concealment mechanism in a suicide staged as homicide.

Hans Gross (1893)	Arthur Conan Doyle (1922)
“*The Investigating Officer observed that on the decayed wooden parapet of the bridge, almost opposite the spot where the corpse lay, there was a small but perfectly fresh injury which appeared to have been caused by the violent blow on the upper edge of the parapet of a hard and angular body*”	“*There was some sign of violence on the stonework of the bridge—a perfectly fresh chip just opposite the body*”
*German original*: Gross (1893), p. 314 (Ref. [2]) *English translation*: Gross (1906), p. 632 (Ref. [3])	*The Problem of Thor Bridge*, *The Strand Magazine*, March 1922, p. 214 (Ref. [4])

**FIGURE 2:**
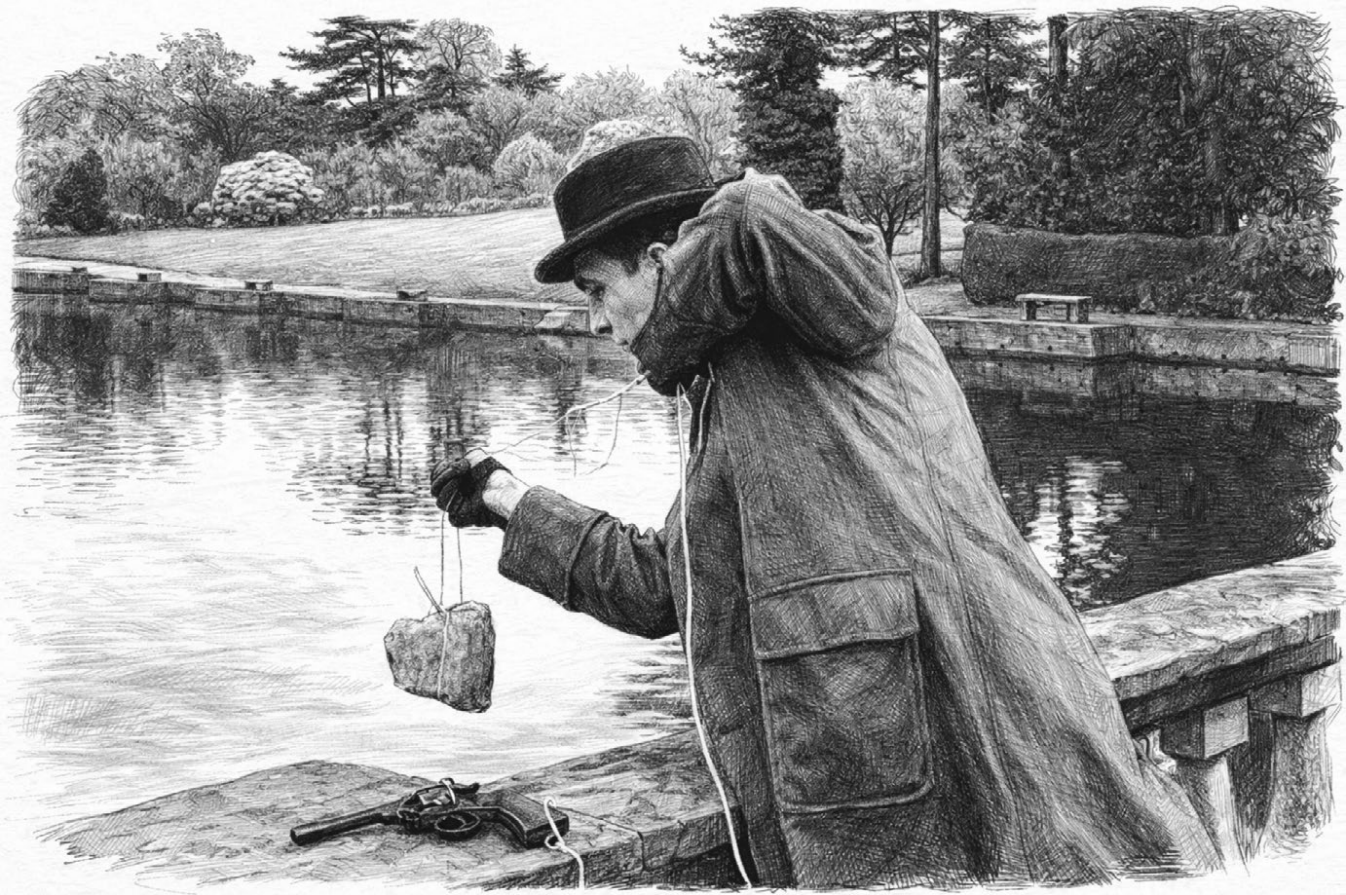
Original drawing based on a film still from *The Problem of Thor Bridge* (1991), part of *The Casebook of Sherlock Holmes* series produced by Granada Television. The drawing depicts Jeremy Brett as Sherlock Holmes examining the setup of the “load‐and‐rope” trick: a revolver tied to a stone and positioned on the bridge parapet, with the cord extending toward the water. This visual dramatization mirrors Hans Gross's 1893 forensic case [2] and illustrates the weapon concealment mechanism later echoed in other suicides staged as homicides and forensic literature [1, 5, 6].

#### 3.2.2 The Greene murder case by S.S. Van Dine

The trick reappears in *The Greene Murder Case* (1928) by S.S. Van Dine [7]. Ada Greene, a mentally unstable character, fakes an alibi by shooting herself and disposing of the weapon using a weighted cord dropped from the window into the snow. Detective Philo Vance discovers a copy of Gross's *Handbuch für Untersuchungsrichter* in her library and quotes the 1893 case in its original German, revealing that Ada had replicated the method with precision.

The 1929 film adaptation of *The Greene Murder Case*, one of the earliest sound films, helped popularize this forensic twist. In a pivotal scene, Philo Vance uncovers a firearm concealed within a wall cavity and tethered to a cord. This cinematic moment represents the first visual dramatization of the “load‐and‐rope” trick—closely mirroring the weapon concealment logic described in Gross's original forensic case [2] and later fictionalized by Conan Doyle in *The Problem of Thor Bridge* [4] (Figure 3).

**FIGURE 3:**
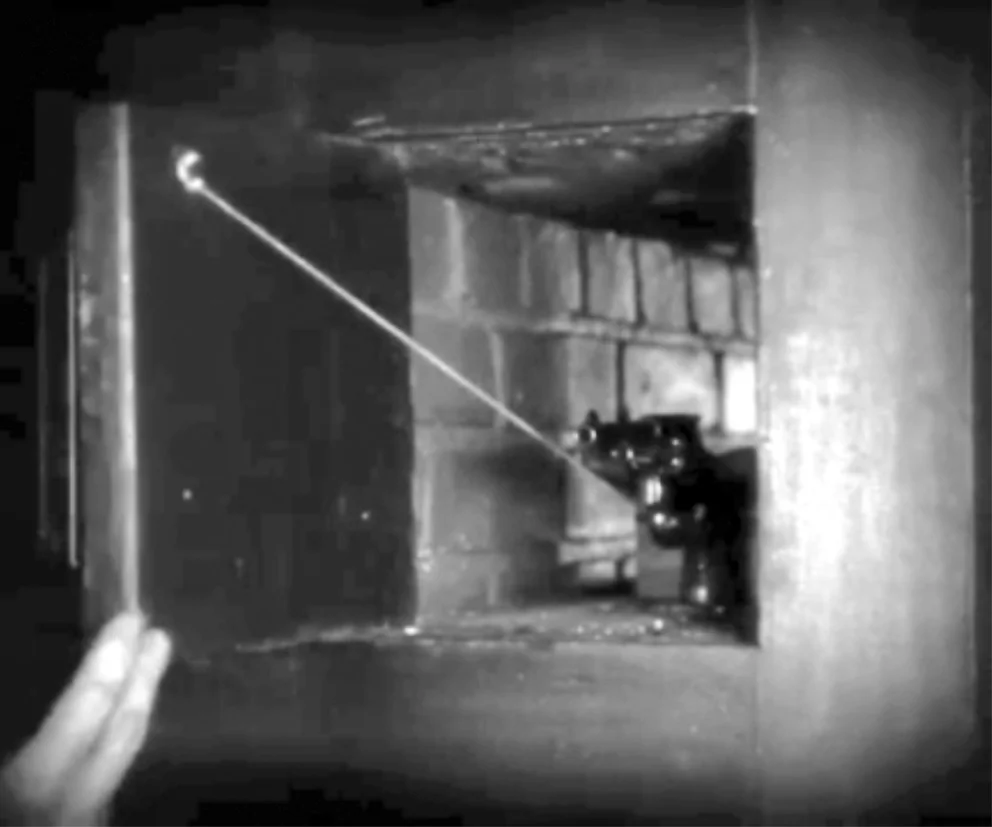
Film still from *The Greene Murder Case* (1929), directed by Frank Tuttle and starring William Powell as Philo Vance. Source: Wikimedia Commons. This work is in the public domain in the United States as it was published before January 1, 1931. The image, captured at minute 58 of the film, depicts the concealed firearm mechanism inside a wall cavity. The setup illustrates the “load‐and‐rope” trick: After discharge, the pistol is retracted into the hiding place via a weighted cord, simulating a weapon concealment. The logic mirrors Hans Gross's original forensic case [2] and its fictional adaptation by Conan Doyle [4].

#### 3.2.3 Detective fiction by George Simenon

In 1929, Georges Simenon reintroduced the “load‐and‐rope” trick into detective fiction, reinforcing its narrative transmission from forensic literature to popular media. Writing under the pseudonym Georges Sim, Simenon published a story featuring Inspector L.53, in which General Yvan Morotzov—a destitute Russian émigré—is found shot dead in a locked and guarded room [8]. No weapon is recovered, and no intruder is detected. Although the scene suggests homicide, it is revealed to be a suicide designed as a homicide to secure insurance benefits for Morotzov's daughter.

To execute the deception, Morotzov ties a silenced revolver to a string weighted by a stone and threads it over a stove pipe. After firing, the weapon is pulled into the pipe and concealed in a stove located in an adjacent room. Inspector L.53 solves the case by identifying minute scratches on the stove pipe, revealing the trajectory of the weapon concealment. The forensic logic mirrors that of Hans Gross's 1893 case [2], suggesting that Simenon, like Conan Doyle, drew inspiration from *Handbuch für Untersuchungsrichter*.

Simenon later reworked the story in *La nuit des sept minutes* (1938), changing the protagonist from Inspector L.53 to Inspector G.7, a reflection of his tendency to recycle narrative structures [9].

In *Monsieur Gallet, décédé* (1931), Simenon revisits the method through Inspector Maigret, who investigates the death of a commercial traveler initially presumed murdered. The victim had in fact committed suicide to defraud an insurance company. Maigret reenacts the “load‐and‐rope” trick in detail, demonstrating his forensic awareness:

“I attach this string to the butt of the weapon… tie this pebble… drop the stone into the well… I shoot at myself… then let go. The stone descends, dragging the revolver… The police arrive, find a corpse, but not the slightest trace of a weapon.” [10]

The novel also introduces a spring‐loaded timer—similar to those used in early self‐photography—that remotely triggers a pistol concealed among trees opposite the window. This appears to be the earliest known reference to such a mechanism in both forensic literature and detective fiction.

Simenon's familiarity with Hans Gross is explicitly acknowledged in *Un crime en Hollande* (1931), where Maigret recommends reading Gross's manuals [10], and in *Les mémoires de Maigret* (1950), where Simenon admits to having read them himself [11].

The recurrence of forensic staging techniques across Simenon's work—combined with their translation into multiple languages and screen adaptations—underscores the porous boundary between forensic history and detective fiction. These narratives helped disseminate the logic of disguising suicides as homicides through weapon concealment, contributing to the cultural transmission of forensic staging strategies.

At difference from The Problem of Thor Bridge, the mechanisms of disguising involved in Van Dine's and Simenon's works are similar to Gross's report but not perfectly the same, probably due to the will of the authors to add complexity and some originality through the lens of their imagination.

### 3.3 From fiction to the crime scene

#### 3.3.1 1970s–1980s

After Gross's report, the first real‐world forensic case of disguised suicide was reported in 1973 by O'Hara in *Fundamentals of Criminal Investigation* [12], which briefly describes a suicide involving a firearm retracted up a chimney by elastic tension, without reporting any additional details. In 1980, Gerdin also reported a case in Sweden involving a retired police superintendent who used a rubber band to propel the weapon into a loft cavity after discharge [13].

These cases appear to be more probably influenced by Van Dine's and Simenon's works, although a specific influential reference cannot be demonstrated by certainty.

#### 3.3.2 1990s–2000s

In the 90s, Gross and Kunz described a Polish case in which a pistol was pulled underwater by a dumbbell [5], and Prahlow et al. reported a US case where a handgun was lowered through a drainage opening to simulate homicide [1]. Both cases were ascribed to imitation of *The Problem of Thor Bridge*.

A further real‐life suicide reported by Durak et al. involved a shotgun rigged to a tree and fired remotely via a 2.5‐meter cord [6]. Although the weapon remained at the scene, the distant‐range wound and absence of residue initially suggested homicide. Only through scene reconstruction and forensic analysis was the death classified as suicide. The mechanical logic of this setup—activating a firearm from a distance—closely parallels the fictional method described in *Monsieur Gallet, décédé* [10], where Simenon's narrative introduces a remotely triggered pistol concealed among trees opposite the window. Both scenarios—Simenon's fictional timer and Durak's real cord mechanism—demonstrate how remote activation can be employed to stage suicide as murder. They reflect a shared forensic staging pattern: the deliberate orchestration of spatial and temporal elements to mislead investigators. Whether imagined in fiction or constructed in reality, such techniques challenge conventional assumptions about weapon proximity and intent.

### 3.4 The “load‐and‐rope” trick on the TV screen

#### 3.4.1 Murder, she wrote

The diffusion of the “load‐and‐rope” trick into mainstream popular culture continued with its appearance in *Murder, She Wrote*, one of the most enduring crime drama series in American television history. In Episode 17 of Season 8, “To the Last Will I Grapple with Thee,” first broadcast on March 15, 1992, Jessica Fletcher investigates the death of Michael O'Connor, who orchestrates his own suicide to resemble homicide [14].

Diagnosed with a terminal illness, O'Connor constructs a complex forensic staging mechanism involving a hidden steel weight and a rope. After firing the weapon, the setup pulls the gun into a wall cavity, creating a weapon concealment scenario designed to implicate his rival, Sean, in a fabricated murder. As Mrs. Fletcher explains in the episode:

“He wasn't murdered. He died at his own hand… Michael O'Connor blamed Sean for all of his problems… So he decided a quick death was preferable over a slow and painful one. And why not take Sean with him?… He cut into a high wall… slipped a heavy steel weight into the gap and attached the other end of the long rope to the gun… as soon as he released the gun, it would be yanked up into the wall.” (Script of the episode, *Murder, She Wrote*, 1992, available at Subslikescript.com. Accessed August 16, 2025)

Unlike Hans Gross's original case, which centered on insurance fraud [2], the motive in this televised adaptation is revenge—closely echoing Conan Doyle's *The Problem of Thor Bridge* [4]. In both narratives, the suicide is designed to frame another individual as murderer, driven by romantic rivalry rather than financial deceit.

These adaptations—from 19th‐century forensic literature to 20th‐century television—demonstrate the enduring appeal and versatility of the weapon concealment trick. They illustrate how Gross's forensic insight has transcended its original scientific context to become a recurring narrative device in crime fiction. Whether in novels, short stories, or screenplays, the “load‐and‐rope” trick continues to captivate audiences and challenge fictional detectives.

Given the widespread reach of these examples, it is reasonable to conclude that fictional transmission has played a significant role in disseminating Gross's forensic staging logic far beyond the readership of his original manual.

#### 3.4.2 Father Brown

*Father Brown* is a fictional Catholic priest created by the British writer G. K. Chesterton, acting as a detective. In 2013, BBC One premiered a television series of Father Brown, presenting a long‐running cycle of episodes based on original plots, characters, and investigative situations.

The mechanical trick of a pistol tied to a rope and counterweight reappeared in Episode 6 of Season 5, *The Eagle and the Daw*, where it becomes the central device around which the entire plot is constructed. The episode was aired for the first time on January 6, 2017 [15].

Father Brown becomes entangled in the case after Katherine Corven, a convicted murderer and one of his long‐standing adversaries, hints from prison that her former lover Raymond Worrall will soon die. When Raymond is later found shot, Father Brown—who had been seen earlier handling Raymond's revolver—is arrested as the prime suspect. With him in custody, Bunty Windermere and Mrs. McCarthy pursue the investigation and uncover the truth: Raymond has engineered his own death as an act of revenge, intending to frame Father Brown for murder and thereby punish him for having previously exposed Katherine's crimes. The reconstruction reveals that Raymond used a pistol tied to a weighted stone, rigged so that once he fired the fatal shot and released the gun, the counterweight would pull it up the chimney, removing the weapon from the scene and creating the illusion of homicide. Bunty's inspection of the rooftop confirms the mechanism: a cord bound to a stone acting as a counterweight, still lodged inside the chimney (Figure 4). Her discovery demonstrates that the gun never left the house but was simply pulled upward and hidden from view, completing Raymond's carefully staged attempt to destroy Father Brown.

**FIGURE 4:**
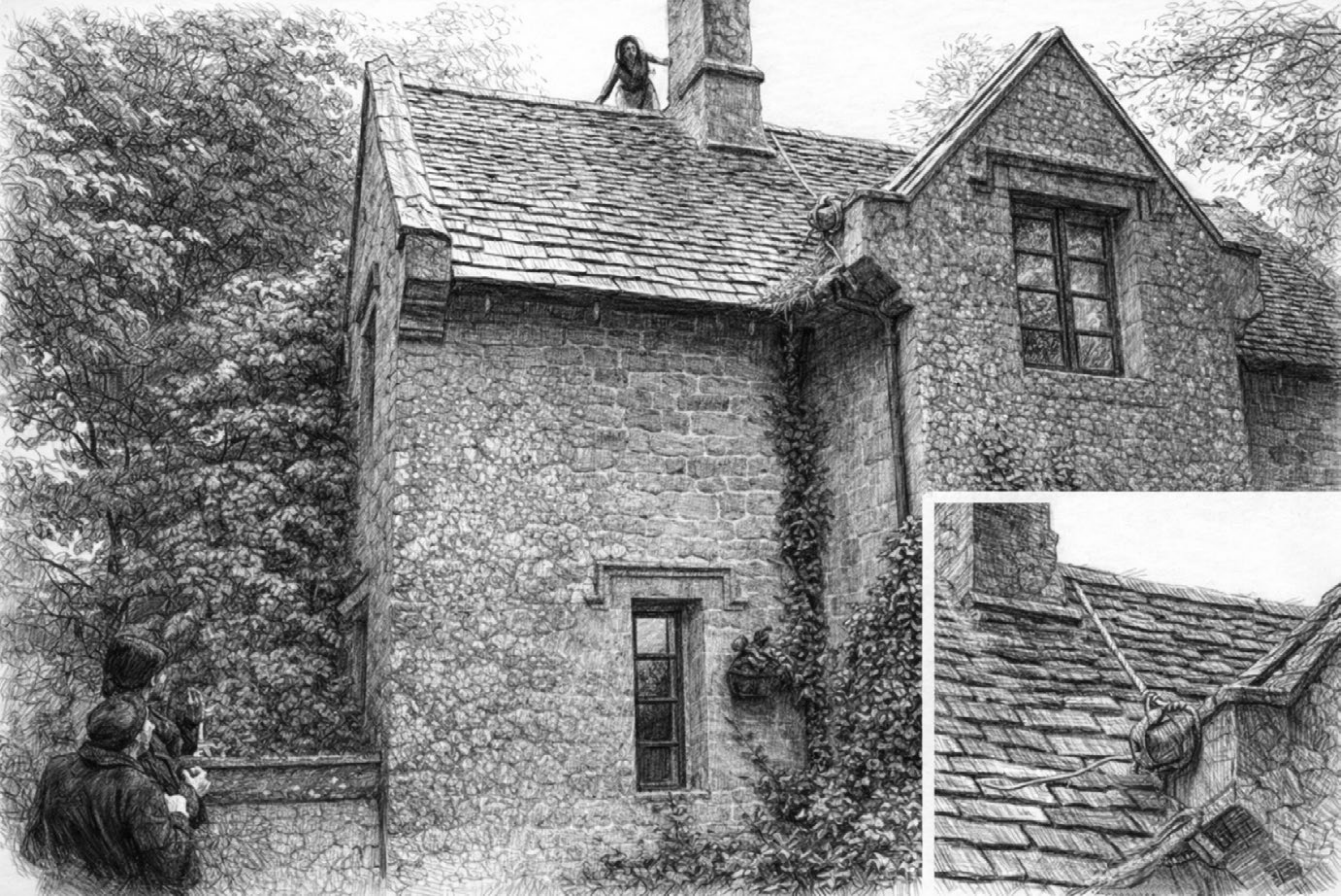
Original drawing based on a film still from the Father Brown episode “The Eagle and the Daw” (2017) [15]. The drawing depicts Penelope “Bunty” Windermere examining the chimney area where the staged‐suicide mechanism had been concealed. The inset provides a close detail of the cord with a stone tied to its end, the counterweight engineered to pull the pistol upward into the chimney immediately after the shot, thereby removing the weapon from the scene and creating the illusion of homicide.

### 3.5 From screen to the crime scene: The helium balloon‐tied gun trick

The “helium balloon‐tied gun” trick represents a compelling instance of fiction shaping forensic reality. In this case, the transmission path is unusually well‐documented: a traceable sequence from televised fiction to real‐life suicide, and subsequently from one forensic case to another via published literature.

The earliest known depiction of this forensic staging method appears in a 2003 episode of *CSI: Crime Scene Investigation*, Season 4, Episode 3, “Homebodies,” where investigators uncover a suicide disguised as homicide [16]. A bounty hunter ties a firearm to helium‐filled balloons, which float away after discharge, removing the weapon from the scene and simulating a textbook murder.

Five years later, the suicide of Thomas Hickman in New Mexico closely mirrors the CSI scenario [17]. Investigators recovered a modified Smith & Wesson revolver tethered to helium balloons, tangled in a nearby cactus. Forensic analysis linked the weapon to metal filings found in Hickman's garage, and digital evidence confirmed prior research into balloon lift capacity and helium purchases. The case was ultimately classified as a suicide staged as homicide, inspired by televised fiction.

In 2018, Alan J. Abrahamson's death in Florida followed a similar pattern [18]. Initially investigated as homicide, the case shifted when police discovered Abrahamson's online search history, including the query “Can you have a gunshot suicide with no weapon present?”—a question that led him to Katherine Ramsland's *The Psychology of Death Investigations* [19], which describes Hickman's case in detail. Investigators found helium tanks, weather balloon calculations, and matching materials at the scene and in Abrahamson's home. The weapon was never recovered, likely carried out over the Atlantic Ocean.

Taken together, these cases illustrate a clear trajectory: a fictional forensic trick imagined in CSI was realized by Hickman in 2008 and later replicated by Abrahamson in 2018. Police investigations in both instances suggest a copycat phenomenon rooted in narrative transmission. The porous boundary between fiction and forensic reality underscores the need for investigators to recognize how cultural narratives may inform criminal staging techniques.

A comprehensive timeline of suicides staged as homicides is presented in Table 2.

**TABLE 2:** Timeline of disguising techniques in suicides staged as homicides: From historical documentation to fictional adaptations and forensic cases.

Source	Year	Staging technique for concealing suicide or intentional self‐harm	Category
Hans Gross: *Handbuch für Untersuchungsrichter als System der Kriminalistik* original case [2]	1893	Load‐and‐rope	Historical documentation
Arthur Conan Doyle: *The Problem of Thor Bridge* [4]	1922	Load‐and‐rope	Fictional adaptation (detective story)
S.S. Van Dine: *The Greene Murder Case* [7]	1928	Load‐and‐rope	Fictional adaptation (detective story)
Georges Simenon: *L.53* [8]	1929	Load‐and‐rope	Fictional adaptation (detective story)
Georges Simenon: *Monsieur Gallet, décédé* [10]	1931	Pistol discharged via remote mechanism	Fictional adaptation (detective story)
Georges Simenon: *La nuit des sept minutes* [9]	1938	Load‐and‐rope	Fictional adaptation (detective story)
O'Hara: *Fundamentals of Criminal Investigation* [12]	1973	Elastic recoil mechanism	Documented Forensic Case
Gerdin: *A Case of Disguised Suicide* [13]	1980	Elastic recoil mechanism	Documented Forensic Case
*Murder, She Wrote* [14]	1992	Load‐and‐rope	Fictional adaptation (TV episode)
Gross & Kunz: *Suicidal shooting masked using a method described in Conan Doyle's novel* [5]	1995	Load‐and‐rope	Documented Forensic Case
Prahlow et al.: *A suicide disguised as a homicide: return to Thor Bridge* [1]	1998	Load‐and‐rope	Documented Forensic Case
*CSI: Crime Scene Investigation*, Season 4, “Homebodies” [16]	2003	Helium balloon‐tied gun	Fictional adaptation (TV episode)
Hickman: suicide inspired by CSI [17]	2008	Helium balloon‐tied gun	Documented Forensic Case
*Father Brown* [15]	2017	Load‐and‐rope	Fictional adaptation (TV episode)
Abrahamson: suicide inspired by Hickman's [18]	2018	Helium balloon‐tied gun	Documented Forensic Case

## 4 DISCUSSION

This overview highlights the recurring use of weapon concealment mechanisms and demonstrates how narrative transmission can shape forensic staging strategies across media and real‐world casework.

The copycat phenomenon refers to the imitation of criminal acts following exposure to prior incidents, often sensationalized through media or fiction [20]. Traditionally applied to homicides—particularly mass shootings and serial killings—copycat behavior involves replication of methods, motives, or symbolic gestures. Psychological traits such as narcissism, psychopathy, and a desire for notoriety are frequently cited as contributing factors, with emotionally charged media coverage acting as a powerful catalyst.

One notable example is the *Dexter*‐inspired homicide committed by Mark Twitchell. The television series *Dexter*, which features a forensic technician who moonlights as a vigilante killer, has been cited in multiple criminal investigations involving mimetic behavior. Twitchell, a Canadian filmmaker, lured his victim using a fake dating profile and recreated elements from the show—including a “kill room”—documenting his actions in a journal titled *SK Confessions*, which opened with the line: “This story is based on true events” [21].

Recent scholarship has expanded the scope of copycat behavior to include suicide emulation. Gualtieri et al. conducted a systematic review showing that media portrayals of suicide—especially involving celebrities or fictional dramatizations—can trigger imitation among vulnerable individuals [22]. The Werther effect, named after Goethe's *The Sorrows of Young Werther*, remains foundational, illustrating how romanticized depictions of suicide can lead to real‐world replication. The study highlights the role of social media, cultural context, and psychological susceptibility.

Our research supports this expanded interpretation by tracing a unique trajectory: a real‐life forensic case—Hans Gross's suicide case with a weapon concealment—was adapted into fictional literature and television, and later re‐emerged in real‐life cases. Fiction does not merely reflect reality; it reshapes it. We hypothesize that this feedback loop—from reality to narrative and back—represents a previously underrecognized mechanism of behavioral transmission in forensic staging.

This dynamic is most clearly demonstrated in the helium balloon suicides. The method first appeared in a 2003 episode of *CSI: Crime Scene Investigation* [16], was emulated by Thomas Hickman in 2008 [17], and later replicated by Alan J. Abrahamson in 2018 [18] after reading about Hickman's case in Katherine Ramsland's *The Psychology of Death Investigations* [19]. The chain of influence—from screen to life, and from one suicide to another—is unusually well‐documented and provides a rare example of narrative transmission with forensic consequences.

A similar, though less direct, pattern is suggested in suicides involving the “load‐and‐rope trick,” popularized in detective fiction by Sir Arthur Conan Doyle [4], S.S. Van Dine [7], and Georges Simenon [8, 9, 10]. These stories introduced forensic staging techniques that later appeared in documented suicides.

It is evident that both Conan Doyle and Simenon drew inspiration from Hans Gross's *Handbuch für Untersuchungsrichter* [2]. Comparative textual analysis of Gross's 1893 case, Doyle's bridge narrative, and Simenon's stove‐pipe scenario reveals clear structural parallels. Van Dine's direct citation of Gross's manual in German further reinforces the interpretation that he deliberately incorporated Gross's forensic framework into his fiction. In contrast, the screenwriters of *Murder, She Wrote* likely derived the trick from fictional sources rather than Gross directly [5].

While it remains impossible to conclusively demonstrate whether real‐life suicides were directly inspired by Gross's writings or their fictional adaptations, the recurrence of identical staging techniques across literature, television, and forensic casework makes such a connection highly probable. Among the documented cases of suicides involving weapon concealment, two stand out for the professional background of the individuals involved—a retired police superintendent [13] and a practicing attorney [1]. Their familiarity with investigative procedures suggests that certain staging methods may derive from prior forensic knowledge. In the case reported by Prahlow et al. [1], the victim/perpetrator also used a plastic bag to prevent positivity to gunshot residue tests. Conversely, little attention was paid in fiction cases in forensic and medico‐legal methods to ascertain firearm distance determination (characteristics of the wound) and hand injuries or gunshot residues. In the case reported by Gerdin [13], the victim's background in law enforcement suggests possible familiarity with forensic manuals such as those by O'Hara [12] and Gross [2]. Gerdin himself speculated that the act may have been a final gesture of complexity aimed at former colleagues. In other instances, such as the cases reported by Gross & Kunz [6] and Prahlow et al. [1], the authors cited Conan Doyle's *The Problem of Thor Bridge* [4] as the most likely source of inspiration—a plausible hypothesis given the widespread cultural diffusion of Sherlock Holmes stories compared with the more specialized forensic texts of Hans Gross. Notably, a television adaptation of *The Problem of Thor Bridge* starring Jeremy Brett aired in 1991, prior to the 1998 suicide described by Prahlow et al., and includes a visual representation of the weapon concealment mechanism (Figure 2), reinforcing the continuity between Gross's forensic case and its fictional reinterpretations.

The enduring cultural presence of Philo Vance and especially Maigret cannot be discounted. These characters have reached vast audiences and may have subtly shaped the forensic imagination of individuals who later enacted suicides disguised as homicides. Furthermore, the cases reported by Gross & Kunz [6] and Prahlow et al. [1] occurred after the broadcast of the *Murder, She Wrote* episode featuring a similar stratagem [5]. The recurrence of the same forensic staging pattern across media makes it difficult to pinpoint a single source. Nonetheless, the convergence of narrative elements strongly suggests a shared lineage. The fact that the Father Brown episode aired in 2017 makes it chronologically impossible for it to have influenced any earlier real‐world suicides. Yet, its deployment of the rope‐and‐counterweight gun mechanism is revealing, because it shows how deeply this device has endured in the cultural imagination of crime fiction. Its reappearance in a modern television drama illustrates how the mechanism continues to circulate in widely consumed popular narratives, remaining intelligible and dramatically effective for a broad contemporary audience.

Several limitations of this study should be acknowledged. First, the qualitative and historical‐comparative nature of the research precludes the establishment of direct causal relationships between fictional sources and real‐world suicides. While the recurrence of identical staging techniques across literature, television, and forensic casework strongly suggests narrative transmission, definitive proof of direct influence remains beyond the scope of this methodology. Second, the corpus relies exclusively on publicly available case reports, forensic manuals, and fictional texts, which may not reflect the full scope of forensic practice or the actual prevalence of suicides staged as homicides. To the best of our knowledge, no additional documented forensic cases involving mechanical weapon concealment in suicides staged as homicides have been reported in the peer‐reviewed literature beyond those included in this study. Undocumented or misclassified cases may exist that would alter the observed patterns. Third, the identification of copycat phenomena is inherently retrospective and subject to confirmation bias, as the selection of sources was guided by the presence of weapon concealment mechanisms. Finally, the cultural and geographic scope of the corpus is limited, with a predominance of Western European and North American sources, which may not capture staging techniques or narrative traditions from other cultural contexts.

Taken together, these cases support the interpretation that fictional forensic techniques, once dramatized in literature and on screen, can permeate public consciousness and influence real‐world behavior [23]. In this paper, we considered a highly specific and rare situation, that is, suicides staged as homicides, which permitted (also for its rarity) the identification of copycat phenomena from literary works and fiction movies/TV series. However, analogous copycat phenomena may be relevant even in other (more common) contexts and their identification may be useful to clarify some forensic aspects. Forensic professionals should remain aware of the potential for narrative transmission to shape criminal and suicidal acts, particularly when staging is involved. This stresses the urgent need for interdisciplinary collaboration among forensic scientists, media scholars, and public health experts for identification and better understanding of possible copycat phenomena.

## CONFLICT OF INTEREST STATEMENT

The authors declare that they have no conflicts of interest relevant to this manuscript.

## Data Availability

Data sharing not applicable to this article as no datasets were generated or analysed during the current study.
